# Applying the biopsychosocial model to unpack a psychosocial support intervention designed to improve antiretroviral treatment outcomes for adolescents in South Africa

**DOI:** 10.11604/pamj.2022.41.166.31985

**Published:** 2022-02-28

**Authors:** Emeka Francis Okonji, Brian Van Wyk, Ferdinand Che Mukumbang

**Affiliations:** 1 School of Public Health, University of the Western Cape, P Bag X17, Bellville, Cape Town 7535, South Africa,; 2 Department of Global Health, University of Washington, Seattle, WA, United State of America

**Keywords:** Adherence and retention, biopsychosocial model, adolescents

## Abstract

Adolescents (10 to 19 years) living with HIV (ALHIV) experience disproportionately poor adherence to antiretroviral treatment (ART) compared to other age groups. Several barriers, including psychosocial challenges, contribute to this observation. Psychosocial support (PSS) interventions show promising results as a strategy to deal with the biological and psychosocial challenges faced by ALHIV. However, there is dearth of information on how psychosocial support interventions designed to improve treatment adherence and retention in care among ALHIV are effective. In this commentary, we used the biopsychosocial model to formulate hypotheses on how the components of a PSS intervention could improve adherence and retention in ART care. Psychological wellbeing, coping strategies, social support, self-efficacy, and disclosure are key components in the intervention designed to improve ART adherence and retention in care. The management of ALHIV for improved ART adherence and retention requires recognising and addressing the complex biological, psychological and social issues peculiar to them.

## Commentary

In 2017, there were approximately 2.1 million adolescents (aged 10 to 19 years) living with HIV globally [1,2], of which more than 90% resided in sub-Saharan Africa [3]. While the overall incidence of HIV amongst adults and children younger than 10 years has declined, HIV incidence amongst adolescents between the ages of 10 and 19 years has increased within this same period [4]. The increase in HIV among adolescents is attributed to the generation of children infected with HIV perinatally, who are surviving into adolescence due to advancements in antiretroviral treatment (ART), and increasing risky (sexual) behaviour exposing adolescents to contracting HIV. Taking 95% or more of prescribed ART is associated with complete viral suppression [5]. Individuals who have lower levels of adherence are at greater risk of morbidity, treatment failure, and developing of drug resistant forms of HIV [6,7], viral progression, and opportunistic infections [8], and further transmission to sexual partners [9].

Several factors have been identified as barriers to ART adherence and retention in care in the general HIV population. Among these barriers are limited access to medications owing to lack of transportation and financial constraints [10]. Food insecurity is associated with non-adherence through two mechanisms: stopping ART when food was unavailable to avoid aggravated (gastrointestinal) side effects, or because taking ART when insufficient food is available increased hunger [11]. Forgetfulness, substance abuse, drug adverse effect, perceived lack of social support, health illiteracy, mental health issues such as depression, self-stigma [7,12,13], and advanced HIV status [14] constitute barriers to ART adherence. Other psychosocial risk factors for poor ART adherence, include being orphaned, changes of guardianship [15], and perception about ART [16]. ART adherence across all populations is highly dependent on complex relationships between the individuals, their families, society and other treatment factors [17]. In addition to the factors affecting ART adherence across all populations, adolescents face additional challenges to adhere to HIV treatment, such as reliance on adults. The ability of ALHIV to successfully transition from reliance on adults to self-managing adherence to medication and attending regular clinic visit is complicated [18]. Many ALHIV have also expressed lack of support regarding how, when and with whom to disclose their HIV status, which can lead to anxiety and depression. Adolescents often face discrimination relating to their risky sexual behaviours and HIV-positive status [12,16]. At the health systems level, issues such as staff shortages, long waiting times, negative experiences with clinic staff and medication stock outs inhibits regular clinic visits and adherence to ART [12,19,20]. Additional health systems barriers include inadequate counselling on medications adherence due to limited clinician patient interaction time and distance to health facility [21].

Furthermore, the transition of HIV from an acute, deadly disease to a manageable chronic disease has enormous implications for the neurocognitive and psychosocial development of children [22]. Studies show that psychosocial disorder impacts negatively on immunological health outcomes [23]. Psychosocial support (PSS) interventions can help ALHIV overcome barriers to adherence and retention in ART care thereby improving immunological health outcomes [24]. However, there are currently limited understanding on how psychosocial support interventions improve adolescents´ ART adherence, retention in care and immunological health outcomes [25]. A recent scoping review showed that individual and group counselling including family-centred group counselling and the use of adolescent treatment supporters were commonly used intervention to improve ART adherence, linkage to care and/or retention in care among adolescents living with HIV [25]. Using the biopsychosocial model [26], we formulated hypotheses to explain how the components of the RTC ALHIV intervention is designed to improve adherence to ART and retain ALHIV in ART to improve health outcomes. Right to Care (RTC) is a South African non-governmental organisation that provides treatment care and support for people living with HIV. This is done through the identification of HIV positive clients, initiating and retaining them on ART care. In this paper, we describe how the adolescent psychosocial support intervention implemented by RTC works to improve adherence and retention in ART care among adolescents living with HIV in Ehlanzeni and Thabo Mofutsanye districts, South Africa, by applying the biopsychosocial model.

**Description of PSS intervention**: RTC developed an adolescent PSS intervention that provides adolescent and youth friendly services (AYFS). A description of the documents that informed the design of the intervention is shown in Annex 1. The intervention employs trained treatment supporters to support adolescents in the age group 10 to 24 years living with HIV (beneficiaries) in adherence and retention in ART care. This is achieved through the use of psychosocial oriented training material known as flipster to facilitate support club session at selected safe spaces with adolescents and young people (i.e. youth clubs, teen clubs, support groups) organized by age (10-13, 14-16, and 17-24-years age groups). The aim of the support club is to produce resilient and empowered adolescents and young people living with HIV who are better informed and better able to make well-informed choices about medication adherence and retention in care. The treatment supporters work with the health facility structures, as they are assisted with the names and addresses of adolescents who have tested for HIV and known to be positive. These treatment supporters with consent from these ALHIV and their caregivers registers them into the RTC adolescents programme where they are guided and counselled on the importance of medication adherence and then regularly followed up. The treatment supporters pre-pack antiretroviral medications for the beneficiaries when they visit the health facility. The flipster topics addresses age specific and sensitive issues that focuses on knowledge about HIV and treatment adherence. Key topics that the intervention seeks to address in order to improve treatment adherence and retention in care are identified and discussed including (1). Biological factors (2). Psychological factors, (3). Social factors. The duration of sessions for each age group varies: 10 to 13 years - 45 minutes; 14 to 16 years - approximately 60 minutes; and 17 years and above -approximately 75 minutes.

**Figure 1:**
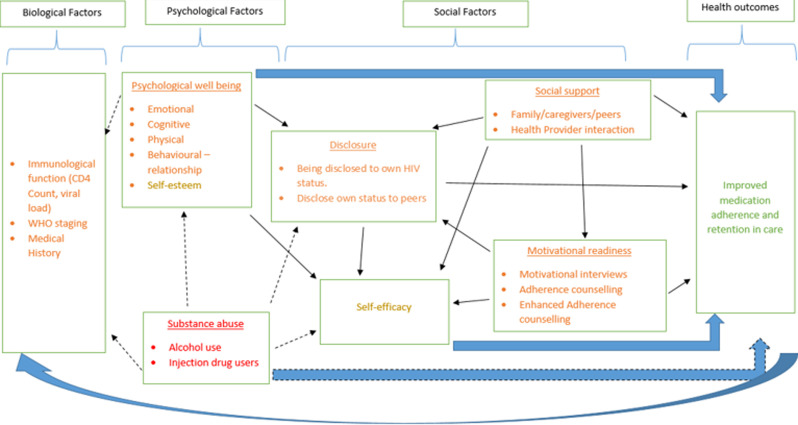
biopsychosocial model explaining the psychosocial support intervention for ALHIV

**Biopsychosocial model for management of HIV among ALHIV**: the biopsychosocial model was originally developed by Engel (1977) who posited that understanding chronic illness and disease as a complex interaction between biological, psychological, and social factors will enable the designing of interventions to improve health outcomes [26]. The biopsychosocial model helps explain the association between the biomedical factors, psychological factors, and social factors at play in the treatment and management of HIV. Therefore, to understand the effectiveness of the RTC´s psychosocial intervention, the bidirectional link between the biological, psychological, social factors and health outcome need to be explored (Figure 1). We choose the biopsychosocial model because we believe that the HIV infection goes beyond biomedical management of HIV to psychological and social management of the impact of the disease to achieve favourable health outcomes. In the following section we describe the biopsychosocial model of illness (i.e. the relationship between the biological, psychological, and social factors) among ALHIV that the PSS intervention addresses towards improving adherence to and retention in ART.

**Biological factors**: the biomedical model assume that disease occurrence is as a result of abnormality of biological molecules inside the body, and excluded the significance of social, psychological and behavioural or social factors of the illness [27]. As such, biological factors relate to elements such as laboratory results (e.g. CD4 count and viral load), symptoms of the illness, and medical history of the patient to determine treatment. It is also noted that several factors such as drug toxicity, being too ill affects medication adherence and retention in care. Similarly, patients with psychological disorder are more likely to experience immunological compromise compared to individuals with no psychological distress. The PSS intervention ensures that ALHIV are screened for TB, as well as weight for age biometrics are taking in order to prescribe a suitable antiretroviral treatment. Furthermore, the PSS intervention improves access to medication access for ALHIV through medication pre-packs and tracking of adolescents who have missed their support group sections. In addition, regular (every six months) viral load monitoring is conducted, and ALHIV who are found to have high viral load are given enhanced adherence counselling section to help them improve their adherence.

**Psychological factors**: psychological wellbeing refers to the absence of depression, anxiety, anger, fear and the presence of positive emotions, meaning, healthy relationships, mastery of one´s environment, engagement, and self-actualization [28]. The intervention addresses stressors that trigger the emotional, cognitive, physical and behavioural aspects of the ALHIV psychological wellbeing through sessions that help them identify situations that can trigger stress and depressive symptoms, and teaching them how to deal with such stressors when they arise. ALHIV face a myriad of emotional, behavioural and mental health challenges such as psychological distress, suicide, and risky sexual behaviours [24]. Similarly, children who experience difficulties in their peer relations have been found to be associated with loneliness [29], which in turn leads to depression and self-perceived stigma and subsequently suicidal particularly for ALHIV.

**Substance abuse**: coping strategies among ALHIV play a role in whether or not patients adhere to their prescribed ART treatments. To cope with stressors, individuals often develop or adopt different coping strategies to help moderate the stressors [30]. Coping strategies can be emotional (e.g. crying, excessive eating, confrontation), avoidance-based (e.g. isolation, mental disengagement, behavioural disengagement, alcohol and other psychoactive drug use, suppression of competing activities) or adaptive strategies (e.g. seeking social support, problem-solving, positive re-appraisal, acceptance, humour, journaling and spirituality) [30]. Evidence suggest that patients who coped by using problem-solving and behaviour-modifying approaches were more likely to be adherent to ART medication compared to those who used emotional or avoidance-based strategies [31]. Drug and alcohol use as a means of coping strategy is associated with non-adherence to ART among people living with HIV (PLHIV) [31]. The PSS intervention seeks to empower ALHIV with problem-solving skills to deal with their HIV status through identifying and dealing with factors that could trigger risky behaviours, as well as to understand the implication of alcohol and drug use along with ART intake. ALHIV were screened for alcohol and drug use, and those found to be using drug or alcohol were provided with enhanced counselling and additional support to help combat alcohol and drug use.

**Self-esteem and self-efficacy**: several sessions that educate on psychological wellbeing, substance abuse, motivational readiness, disclosure, and social support are posited to have direct impacts on self-esteem and self-efficacy. Furthermore, the intervention provides ALHIV with the skill to alter negative responses as well as understand the benefits of sharing positive feedback with peers. For example, ALHIV are skilled to recognise the concept of feelings and learning to speak about feelings. Self-efficacy is widely documented as an important correlate of medication adherence in the treatment of HIV [32,33]. However, positive health care provider interactions may foster greater adherence self-efficacy, which is associated with better adherence to medications [34]. Furthermore, self-efficacy and reduced psychological distress were significantly correlated with adherence [35]. The sessions in the flipster support group utilises an approach that enables ALHIV to understand their emotions and underlying patterns of behaviour. By talking through these emotions and behaviours with a social worker, ALHIV come to know themselves better and make better decisions for themselves.

**Disclosure**: the intervention provides disclosure training at two levels. Firstly, clinicians guide parents or caregivers to disclose HIV status to the adolescents. Secondly, uses role plays in dyads to help ALHIV understand the importance of disclosing their own HIV status as well as guiding them through the process of disclosure and overcoming challenges relating to disclosure. Having disclosed to peers was significantly related to regular visits to the HIV clinic, and greater social support through peers. ALHIV need safe environments to practice disclosure skills [36]. Interventions should enable them to make optimal use of available psychosocial resources even under constraining conditions such as disruptive family structures [36], including ALHIV who are orphans and their caregivers [37]. Another study that investigated path analysis of disclosure revealed that disclosure to family members had significant indirect effects on adherence via social support and self-efficacy [38].

**Social support**: the intervention utilises peers and dedicated clinicians to provide HIV treatment care and support for ALHIV thereby strengthening the relationship between treatment supporters/clinicians and ALHIV. In addition, parents/caregivers of ALHIV are provided with training on how to support the ALHIV in their treatment journey. Therefore, the network of support provided to ALHIV is designed to improve their adherence and retention in ART care. Studies have suggested that ALHIV who receive support from clinicians, peers, and/or caregivers improve their psychosocial wellbeing, which in turn improves adherence and retention in ART [33,39,40]. Another study found social support specific to taking medications was correlated with self-efficacy [35]. Engagement with health care providers (HCP) includes access to HCP as needed, information sharing, involvement of client in decision making and self-care activities, respect and support of the HCP for the client´s choices, and management of client concerns. Promoting engagement with the HCP is necessary to facilitate skills that help PLHIV manage their HIV [41]. Improving the HIV treatment-related knowledge and self-efficacy of caregivers may help to improve the clinical outcomes of HIV-infected children [42].

**Motivational readiness**: motivational interviewing (MI) addresses patient ambivalence about a desired goal in a directed, patient-centred manner. MI intervention is established as a therapeutic tool within the pediatric population with positive outcomes for obesity, asthma, medication adherence and HIV management. MI is especially promising within the adolescent population where increasing independence tends to contribute to poorer health outcomes [43]. MI may be a promising intervention for adolescents and young adults (AYAs) with chronic illness in addressing non-adherence and potentially improving quality of life [44]. MI appears to be a promising intervention to improve HAART adherence in HIV-positive individuals [45], reductions in viral load (in the short term) and unprotected sexual acts, and a reduction in alcohol use was identified only in one of two studies that reported on this outcome. Retention rates were not affected by the intervention [46]. The intervention utilises this approach to initiate ALHIV into the ART programme as well as, provide enhanced adherence counselling to ALHIV with virologically failure to enable them adhere to ART.

## Conclusion

The management of ALHIV in relation to ART adherence and retention requires identifying the complex developmental issues peculiar to this particular age and addressing the various components that inhibits optimal adherence to ART and retention in care. The psychosocial support club provides a compendium of topics that addresses the various developmental issues in this particular age group.

## Annex

**Annex 1**: a description of the documents that were included in the document review
